# Detection of central nervous system viral infections in adults in Manado, North Sulawesi, Indonesia

**DOI:** 10.1371/journal.pone.0207440

**Published:** 2018-11-16

**Authors:** Arthur H. P. Mawuntu, Janno B. B. Bernadus, Rama Dhenni, Ageng Wiyatno, Riane Anggreani, Feliana, Frilasita A. Yudhaputri, Ungke Anton Jaya, Chairin Nisa Ma’roef, Aghnianditya K. Dewantari, Araniy Fadhilah, Jeremy P. Ledermann, Ann M. Powers, Dodi Safari, Khin Saw Aye Myint

**Affiliations:** 1 Faculty of Medicine, Sam Ratulangi University, Manado, Indonesia; 2 Eijkman Institute for Molecular Biology, Jakarta, Indonesia; 3 Eijkman-Oxford Clinical Research Unit, Eijkman Institute for Molecular Biology, Jakarta, Indonesia; 4 Division of Vector-Borne Diseases, Centers for Disease Control and Prevention, Fort Collins, Colorado, United States of America; Cincinnati Children's Hospital Medical Center, UNITED STATES

## Abstract

Central nervous system (CNS) viral infections are important causes of morbidity and mortality worldwide but the systematic survey of patients admitted to hospitals with CNS infections in many countries, including Indonesia, is limited. To obtain more information regarding the causes of CNS infections in Indonesia, this study was performed to detect and identify viral agents associated with CNS infections amongst in-patients at a referral hospital in Manado, North Sulawesi, Indonesia. Adult patients admitted to R.D. Kandou General Hospital with presumed CNS infection were enrolled. Cerebrospinal fluid, serum, and throat swab samples were collected and tested using molecular, serological, and virus isolation assays. A confirmed viral etiology was established in three and a probable/possible in 11 out of 74 patients. The most common was herpes simplex virus 1 (7/74, 9.5%), followed by Epstein-Barr virus (2/74, 2.7%), cytomegalovirus (1/74, 1.4%), enterovirus D68 (1/74, 1.4%), rhinovirus A (1/74, 1.4%), dengue virus (1/64, 1.6%), and Japanese encephalitis virus (1/64, 1.6%). There were 20 fatal cases (27.0%) during hospitalization in which eight were associated with viral causes. We identified herpes simplex virus 1 as the most common cause of CNS infection among adults in North Sulawesi with most of the cases remaining undiagnosed. Our study highlights the challenges in establishing the etiology of viral CNS infections and the importance of using a wide range of molecular and serological detection methods to identify CNS viruses.

## Introduction

Central nervous system (CNS) infections including meningitis and encephalitis are important causes of significant mortality and morbidity in the developing nations. Viruses are considered as important etiological agents of CNS infections, causing diseases ranging from febrile illness to myelitis to meningoencephalitis [1]. However, in most cases, the etiology of CNS infection is not known due to lack of diagnostic capacity, standard clinical case definitions, or low levels of surveillance. Specific diagnosis for CNS infection is rarely made and usually categorized empirically as only “viral” or “bacterial”. There have been few reports on the viral etiologies of CNS infections in Indonesia except for Japanese encephalitis virus (JEV), a leading cause of acute encephalitis in children and young adults in the Southeast Asian region [2–5]. Still, JEV is significantly underreported in Indonesia. Furthermore, in endemic provinces like Bali where encephalitis is often suspected to be JEV [5], there is lack of laboratory capability to accurately determine the disease burden of JEV and other CNS viruses. The objective of this study was therefore to detect and identify the pathogens responsible for viral CNS infections amongst in-patients at a referral hospital in Manado, North Sulawesi, Indonesia.

## Materials and methods

### Ethical approval

This study was approved by the Medical Research Ethics Committee of R.D. Kandou General Hospital (Ethical Approval No. 066/EC-UPKT/III/2016) and Eijkman Institute Research Ethics Commission (Ethical Approval No. 78). Written informed consent for participating in the study was obtained from all of the patients, guardians, or accompanying close relatives.

### Patients, clinical data, and samples collection

The study was conducted at R.D. Kandou General Hospital, Manado, North Sulawesi, a tertiary referral hospital at the northeastern part of Indonesia that occasionally receives patients from other provinces including Central Sulawesi, North Sulawesi, North Maluku, Maluku, West Papua, and Papua. Patients (age ≥15 years) presenting at the adult wards from August 2015–February 2017 with presumed CNS infections were enrolled based on the following criteria: acute onset (≤7 days) fever (>37.5°C) with altered mental state, accompanied by seizures, or focal neurological findings or neck stiffness. Blood and/or cerebrospinal fluid (CSF) samples were collected along with computed tomographic (CT) scan with and without contrast as part of their assessment. In addition, throat swabs were also collected from those without altered consciousness, agitation or seizures, and not critically ill, in viral transport medium containing Eagle’s minimum essential medium, 500 U/ml penicillin, 500 μg/ml streptomycin, and 10% fetal bovine serum (Gibco, Carlsbad, CA). The specimens were stored in liquid nitrogen until required for further testing.

Demographic and laboratory data, including routine hematology and CSF biochemistry, data questionnaire regarding illness, occupation, animal exposure, and human immunodeficiency virus (HIV) and tuberculosis (TB) status were collected on case record forms at enrollment and/or during hospitalization. History of illness and symptoms were obtained from guardians, or accompanying close relatives if the subject was unable to communicate. CSF was examined by lateral flow assay and India ink for detection of *Cryptococcus neoformans*, Gram stain for detection of bacteria, and direct acid-fast bacilli smear examination for detection of *Mycobacterium tuberculosis*. In addition, chest X-ray was also performed if clinically indicated for TB. With limited capacity for advanced microbiological testing, CSF bacterial and TB cultures were not performed on-site. Undiagnosed CNS infection patients regardless of the HIV status, TB status, and CSF laboratory results were enrolled to determine the viral cause and HIV-associated viral infections. In addition, all undiagnosed CNS infections regardless of the presumptive clinical diagnosis (meningitis/encephalitis) were included in this study. Clinical outcome was scored on discharge according to the Glasgow Outcome Scale (GOS) [6]. Patients were monitored at the hospital longitudinally until discharge or death.

An integrated approach using molecular, serological, and virus isolation assays was performed retrospectively on collected specimens at the Eijkman Institute for Molecular Biology, Jakarta. The CNS infection was classified as confirmed, probable, or possible based on each individual pathogen, type of specimen, association with previous medical condition, and clinical characteristics [7]. In brief, etiology was considered confirmed for cases with positive results by molecular tests or virus isolation or virus-specific IgM in CSF for a pathogen considered to be a well-established cause of encephalitis. For example, detection of herpes simplex 1 (HSV-1) and cytomegalovirus (CMV) DNA in CSF was considered confirmatory. Etiology was considered probable if 1) the pathogen or its nucleic acid were not detected in CSF but there was serological evidence or 2) in cases where a pathogen not generally causing encephalitis was detected in CSF. For example, detection of anti-JEV IgM in a single serum sample was considered probable. Etiology was considered possible for cases with positive results other than in CSF with evidence of acute infection that suggested a potential etiologic role in CNS infection. For example, detection of HSV-1 and enteroviruses (EV) by PCR/RT-PCR in serum or throat swab samples were considered possible.

### Nucleic acid extraction

Viral nucleic acids were extracted using QIAamp Viral RNA Mini kit (Qiagen, Hilden, Germany) which extracts both DNA and RNA. The procedure was conducted according to the manufacturer’s instruction. Elution volume was 60 μl.

### PCR and RT-PCR assays

Conventional PCR or RT-PCR was used to detect alphaviruses [8], flaviviruses [9], filoviruses [10], paramyxovirus [11], coronaviruses [12,13], herpesviruses [14], and enteroviruses [15], using broadly reactive group primers. These PCR/RT-PCR panels cover a broad range of known neurotropic human viruses including but not limited to chikungunya (CHIKV), Japanese encephalitis (JEV), dengue (DENV), West Nile (WNV), measles (MV), Nipah (NiV), human coronavirus OC43 (HCoV-OC43), herpes simplex virus 1 and 2 (HSV-1 and HSV-2), cytomegalovirus (CMV), Epstein-Barr (EBV), human herpesvirus 6, enterovirus 71 and D68 (EV-71 and EV-D68), and rhinovirus A (RV-A). All primers have been previously described in previous publications [8–16]. For alphavirus and flavivirus RT-PCR, Qiagen OneStep RT-PCR (Qiagen) kit was used. For all other PCR or RT-PCR, GoScript Reverse Transcription System and GoTaq Green Master Mix kits were used (Promega, Madison, WI, USA). Synthetic DNA plasmid constructs kindly provided by PREDICT USAID were used as ‘universal positive controls’ for filovirus, paramyxovirus, coronavirus, herpesvirus, and enterovirus PCR as described before [16]. RNA extracted from isolates of JEV Nakayama strain and CHIKV 181/25 strain were used for flavivirus and alphavirus RT-PCR controls, respectively. Extracted viral nucleic acid from available CSF, serum, and throat swabs were amplified by PCR/RT-PCR panels described above, from which positive amplicons were further purified and sequenced with both forward and reverse primers using Big Dye Terminator v3.1 Ready Reaction Mix (Applied Biosystems, Carlsbad, CA, USA). Sequences were analysed by BLAST analysis to identify each virus to species.

All the PCR/RT-PCR assays used in this study were designed to detect both known human pathogen and potentially novel viruses, facilitated by the use of degenerate primers, multiplex primers, and/or nested two-step PCR format with sequencing of the PCR product as the confirmatory step. Although the non-arboviral group primers have not been evaluated with clinical specimens, the analytical sensitivities have been reported elsewhere [8–16]. In addition, in-house validation of the PCR/RT-PCR assays were performed by testing each panel against extracted RNA from serial dilutions of known virus isolates or synthetic DNA control. The pan-flavivirus RT-PCR was able to detect extracted RNA from at least 0.4 PFU/ml of DENV-1, -2, -4; 400 PFU/ml of DENV-3; 0.4 PFU/ml of JEV; and 4 PFU/ml of ZIKV; while the pan-alphavirus RT-PCR was able to detect 1,000 PFU/ml of CHIKV 181/25 strain. The flavivirus isolates used to evaluate pan-flavivirus RT-PCR have been described elsewhere [17]. The pan-enterovirus RT-PCR was able to detect at least 12 PFU/ml poliovirus Sabin vaccine strain and 89 copies of synthetic DNA control. The pan-paramyxovirus RT-PCR was able to detect 1.4 PFU/ml measles virus vaccine strain CAM70 and 2.5 copies of synthetic DNA control. Finally, the pan-filovirus, pan-coronavirus, and pan-herpesvirus PCR/RT-PCR were able detect at least 60 copies, 28 copies, and 1 copy of synthetic DNA control, respectively. Representative gel electrophoresis with positive results from the PCR/RT-PCR is shown in S1 Fig.

### Serology assays

Commercial IgM ELISA test kits were used on acute-phase sera and CSF to detect DENV-specific antibody (Panbio Dengue IgM Capture ELISA, Alere, Waltham, MA, USA) and JEV-specific antibody (JE Detect IgM Antibody Capture ELISA, InBios, Seattle, WA, USA). The tests were performed and interpreted following manufacturers’ recommendations.

### Virus isolation

CSF specimens were inoculated onto African green monkey kidney Vero cells as previously described [18]. Cells were observed daily for cytopathic effect.

### Statistical analysis

Quantitative data differences between two groups were compared by Mann-Whitney test and categorical data were compared using Fisher’s exact test. *P*-values less than 0.05 were considered significant.

## Results

**Fig 1:**
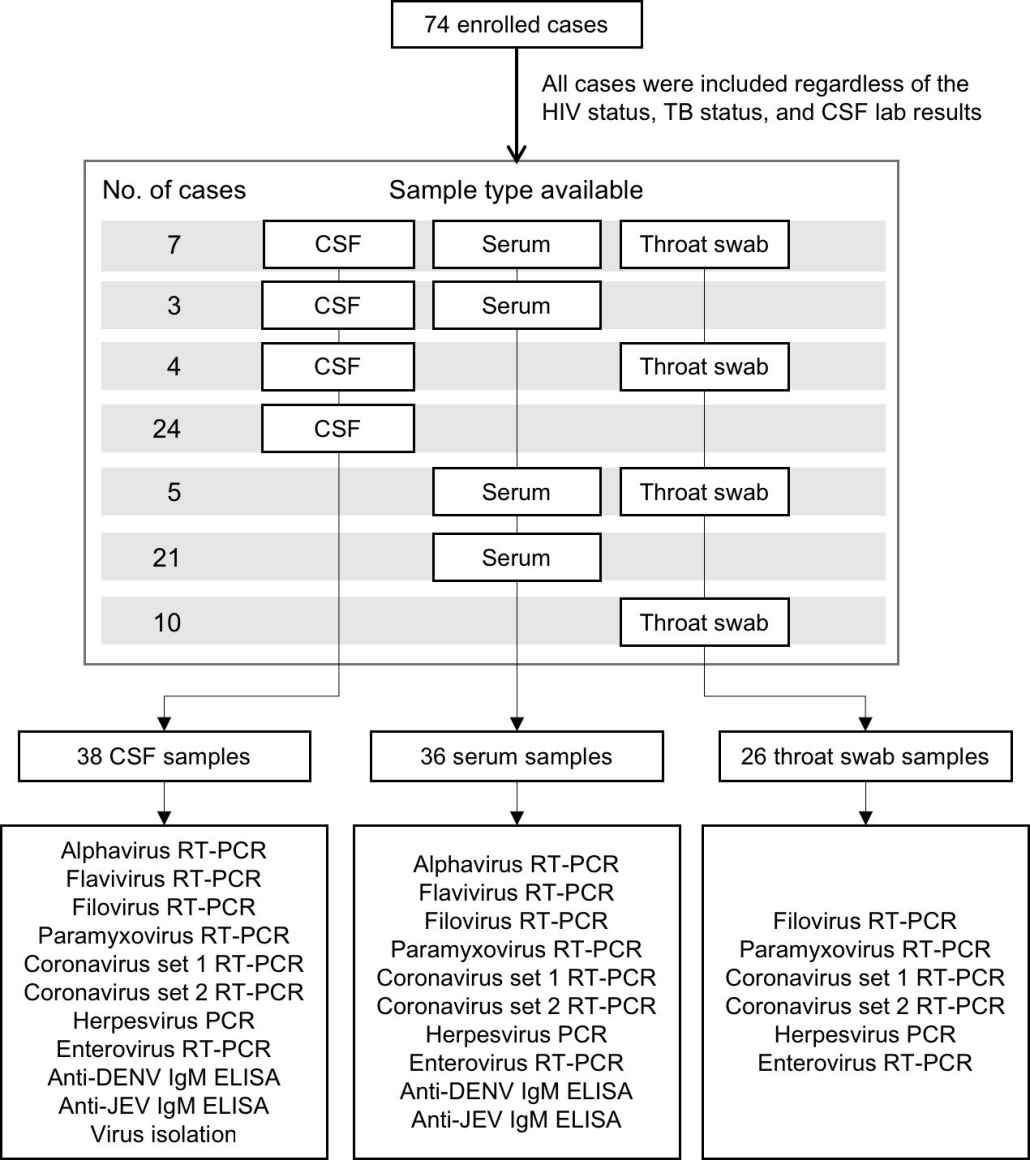
Flow diagram for total enrolled cases, collected samples available, and assays performed for different sample types in the study.

A total of 74 patients, all from North Sulawesi, were enrolled with a clinical suspicion of CNS infection during the 18-month study period (Fig 1). Details of clinical features, laboratory investigations, and clinical outcomes are presented in Table 1. Forty-nine of the patients (66.2%) were male with a median age of 31 years (range 15–72). Median illness day at admission was 7 days (range 1⎼90), while the median duration of hospital stay was 12.5 days (range 2⎼51). Median duration of hospital stay was 11.5 (range 4⎼38) for deceased patients and 12.5 days (range 2⎼51) for those survived. Twenty-three patients (31.1%) were HIV-positive, while 33 (44.6%) had TB from previous medical history. Most patients (73.0%) had a high-grade fever on admission as well as headache (67.6%), altered consciousness (74.3%), or seizures (37.8%). CT scan results were abnormal in 68.9% of patients (42 out of 61) mostly with meningeal enhancement and focal brain lesions in 36.7% and 20.0% of the patients, respectively.

**Table 1:** Characteristics of adult patients with suspected CNS infection enrolled in this study.

Parameter			All cases n = 74^a^	Diagnosed n = 14^a^	Undiagnosed n = 60^a^
**Clinical**					
	Age, median (range)		31 (15⎼72)	30 (15⎼68)	31 (15⎼72)
	Sex, male		49 (66.2)	7 (50.0)	42 (70.0)
	High grade fever (axillary temperature ≥39°C)		54 (73.0)	9 (64.3)	45 (75.0)
	Day of illness at admission, median (range)		7 (1⎼90)	6 (1⎼30)	7 (1⎼90)
	Duration of hospital stay, median (range)		12.5 (2⎼51)	11 (5⎼38)	12.5 (2⎼51)
		Survived patients	12.5 (2⎼51)	11.5 (7⎼15)	12.5 (2⎼51)
		Deceased patients	11.5 (4⎼38)	10.5 (5⎼38)	12.5 (4⎼30)
	Headache		50 (67.6)	7 (50.0)	43 (71.7)
	Seizures		28 (37.8)	7 (50.0)	21 (35.0)
	Altered consciousness		55 (74.3)	13 (92.9)	42 (70.0)
	Skin rash		7 (9.5)	2 (14.3)	5 (8.3)
	Myalgia		11 (14.9)	3 (21.4)	8 (13.3)
	Focal neurologic signs		29 (39.2)	4 (28.6)	25 (41.7)
	Glasgow Coma Scale, median (range)		13 (3⎼15)	10.5 (3⎼15)^c^	13.5 (7⎼15)
	Cognitive impairment		7/67 (10.5)	3/14 (21.4)	4/53 (7.6)
	HIV-positive status		23 (31.1)	5 (35.7)	18 (30.0)
	TB-positive history status		33 (44.6)	4 (28.6)	33 (55.0)
	Cryptococcus-positive		6 (8.1)	0 (0.0)	6 (10.0)
**Laboratory Investigations**					
	CSF total cell count, cells/μl, median (range)^b^		5 (0⎼3,750)	5 (0⎼200)	5 (0⎼3,750)
	CSF mononuclear cell count, cells/μl, median (range)^b^		20 (0⎼100)	10 (0⎼95)	20 (0⎼100)
	CSF polymorphonuclear cell count, cells/μl, median (range)^b^		2 (0⎼90)	2.5 (0⎼80)	2 (0⎼90)
	CSF protein mg/dl, median (range)^b^		40 (10⎼1,730)	120 (20⎼730)	40 (10⎼1,730)
	CSF glucose mg/dl, median (range)^b^		62 (5⎼135)	68 (33⎼135)	58 (5⎼128)
	CSF/serum glucose ratio <0.4^b^		7 (18.0)	0 (0.0)	7 (20.6)
	Leukocytosis		39 (52.7)	7 (50.0)	32 (53.3)
	Leukopenia		3 (4.1)	0 (0.0)	3 (5.0)
	Thrombocytosis		4 (5.4)	2 (14.3)	2 (3.3)
	Thrombocytopenia		5 (6.8)	1 (7.1)	4 (6.7)
	Elevated ALT		17/70 (24.3)	6/14 (42.9)	11/56 (19.6)
	Elevated AST		33/70 (47.1)	9/14 (64.3)	24/56 (42.9)
**Neuro-Imaging**					
	CT scan, abnormal		42/61 (68.9)	5/9 (55.6)	37/52 (71.2)
	Meningeal enhancement		22/60 (36.7)	2/9 (22.2)	20/51 (39.2)
	Focal brain lesions		12/60 (20.0)	1/9 (11.1)	11/51 (21.6)
**Outcome at Discharge**					
	Death (GOS = 1)		20 (27.0)	8 (57.1)^d^	12 (20.0)
	Persistent vegetative state (GOS = 2)		12 (16.2)	0 (0.0)	12 (20.0)
	Severe disability (GOS = 3)		7 (9.5)	1 (7.1)	6 (10.0)
	Moderate disability (GOS = 4)		15 (20.3)	4 (28.6)	11 (18.3)
	Low disability (GOS = 5)		20 (27.0)	1 (7.1)	19 (31.7)
	Neurological sequelae		20/54 (37.0)	0/6 (0.0)	20/48 (41.7)
	Cognitive sequelae		16/54 (29.6)	2/6 (33.3)	14/48 (29.2)

Data are presented as number of patients (%) unless indicated otherwise. HIV, human immunodeficiency virus; TB, Tuberculosis; ALT, alanine transaminase; AST, aspartate transaminase; GOS, Glasgow Outcome Score.

^a^n as in column heading unless otherwise specified in individual rows. Percentage noted in parentheses unless otherwise noted.

^b^Data available from 39 patients (6 diagnosed and 33 undiagnosed).

^c^*P*-value < 0.01 when comparing patients with identified etiology to those undiagnosed.

^d^*P*-value < 0.05 when comparing patients with identified etiology to those undiagnosed.

Only 38 patients who gave consent for lumbar puncture had CSF available in sufficient quantities for viral testing, while 36 patients had only serum and/or throat swabs (Fig 1). The CSF and serum specimens were screened with two arbovirus (alphavirus and flavivirus) and six non-arbovirus degenerate primer sets and by two serological assays (anti-DENV IgM and anti-JEV IgM). In addition, throat swab samples were tested for the six non-arbovirus panels. The viral diagnostic assays performed and viruses identified were summarized in Table 2. A confirmed viral etiology was identified in three (HSV-1, CMV, DENV) (4.1%) and a probable/possible association in 11 (HSV-1, JEV, EBV, EV-D68, RV-A) (14.9%) of enrolled patients. Twelve cases were identified by PCR/RT-PCR while two were identified by serology. The most common viral agent HSV-1 was identified in seven patients (9.5%). Other viruses found were EBV (n = 2, 2.7%), CMV (n = 1, 1.4%), EV-D68 (n = 1, 1.4%), RV-A (n = 1, 1.4%), DENV (n = 1, 1.6%), and JEV (n = 1, 1.6%). There were no viruses isolated from any of the CSF samples. Patients with identified viral infection were found to have lower GCS (median 10.5 [3⎼15] vs 13.5 [7⎼15], *P* < 0.01) and higher fatality (57.1% vs 20.0%, *P* < 0.05) than those undiagnosed (Table 1).

**Table 2:** Viral diagnostic assays and detection from adult patients with suspected CNS infection.

Virus diagnostic assays			Number of samples, positive/tested			Number of patients, positive/tested (%)	Number of patients with CNS viral infection diagnosis		
			CSF	Serum	Throat Swab		Confirmed	Probable	Possible
**Molecular**									
	Alphavirus		0/38	0/36	ND	0/64 (0)	0	0	0
	Flavivirus		0/38	0/36	ND	0/64 (0)	0	0	0
	Filovirus		0/38	0/36	0/26	0/74 (0)	0	0	0
	Paramyxovirus		0/38	0/36	0/26	0/74 (0)	0	0	0
	Coronavirus set 1^a^		0/38	0/36	0/26	0/74 (0)	0	0	0
	Coronavirus set 2^a^		0/38	0/36	0/26	0/74 (0)	0	0	0
	Herpesvirus								
		HSV-1	1/38	1/36	5/26	7/74 (9.5)	1	0	6
		EBV	0/38	0/36	2/26	2/74 (2.7)	0	0	2
		CMV	1/38	0/36	0/26	1/74 (1.4)	1	0	0
	Enterovirus								
		EV-D68	0/38	0/36	1/26	1/74 (1.4)	0	0	1
		RV-A	0/38	0/36	1/26	1/74 (1.4)	0	0	1
**Serology**									
	Anti-DENV IgM		1/38	1/36	ND	1/64 (1.6)	1	0	0
	Anti- JEV IgM		0/38	1/36	ND	1/64 (1.6)	0	1	0
**Virus isolation**			0/38	ND	ND	0/38 (0)	0	0	0

HSV-1, herpes simplex virus 1; EBV, Epstein-Barr virus; CMV, cytomegalovirus; EV-D68, enterovirus D68; RV-A, rhinovirus A; DENV, dengue virus; JEV, Japanese encephalitis virus; ND, not done.

^a^Coronavirus set 1 RT-PCR panel was adapted from [12], while coronavirus set 2 was adapted and modified from [13] which can detect broader range of bat coronavirus strains.

In our study with a small sample size, no distinguishable clinical or laboratory characteristics were observed between the different viral agents (Table 3). Five of eight deaths were associated with HSV-1 infection while the remaining three were associated with EBV, CMV, and JEV infections. One HSV-1 and two EBV cases were both HIV-positive and TB history-positive, while one HSV-1 and EV-D68 were HIV-positive only.

**Table 3:** Clinical and laboratory characteristics of patients with identified viral infection.

No.	Viral etiology diagnosis	HIV status	TB history	Outcome^a^	Age, sex	CSF laboratory				CT scan	Clinical characteristics								
						Cell count, cell/μl	Protein, mg/dl	Glucose, mg/dl	CSF/ serum glucose ratio		High grade fever ≥39°C (days)	Seizure (days)	Headache (days)	Altered consciousness (days)	Skin rash (days)	Myalgia (days)	Focal neurologic signs	GCS^b^	Cognitive impairment
1	Confirmed HSV-1	⎼	⎼	Low disability	68, M	200	170	62	0.4	Abnormal	+ (2)	⎼	+ (7)	+ (2)	⎼	⎼	⎼	12	⎼
2	Possible HSV-1	⎼	⎼	Death	21, F	NA	NA	NA	NA	Abnormal	⎼	⎼	+ (9)	+ (7)	⎼	⎼	⎼	3	+
3	Possible HSV-1	⎼	⎼	Moderate disability	38, M	NA	NA	NA	NA	NA	+ (4)	⎼	+ (4)	+ (1)	⎼	+ (3)	⎼	8	⎼
4	Possible HSV-1	⎼	+	Death	57, F	NA	NA	NA	NA	Normal	+ (7)	⎼	⎼	+ (4)	⎼	⎼	⎼	11	⎼
5	Possible HSV-1	⎼	⎼	Death	15, F	0	70	71	0.6	Abnormal	+ (5)	⎼	⎼	+ (2)	⎼	⎼	+	9	⎼
6	Possible HSV-1	+	+	Death	37, M	10	240	33	0.5	NA	⎼	+ (1)	+ (3)	+ (3)	⎼	⎼	+	9	⎼
7	Possible HSV-1	+	⎼	Death	31, M	NA	NA	NA	NA	NA	⎼	⎼	+ (14)	+ (3)	+ (30)	⎼	+	14	+
8	Possible EBV	+	+	Death	20, F	NA	NA	NA	NA	Abnormal	+ (3)	+ (2)	+ (14)	+ (14)	⎼	+ (6)	+	9	+
9	Possible EBV	+	+	Moderate disability	29, M	NA	NA	NA	NA	Normal	+ (2)	⎼	⎼	+ (2)	⎼	⎼	⎼	11	⎼
10	Confirmed CMV	⎼	⎼	Death	15, F	0	38	135	1.0	Normal	+ (5)	+ (7)	⎼	+ (5)	⎼	⎼	⎼	10	⎼
11	Possible EV-D68	+	⎼	Moderate disability	29, M	NA	NA	NA	NA	Abnormal	⎼	+ (30)	⎼	⎼	⎼	⎼	⎼	15	⎼
12	Possible RV-A	⎼	⎼	Moderate disability	39, F	0	730	77	0.6	Normal	⎼	+ (4)	+ (7)	+ (1)	⎼	⎼	⎼	14	⎼
13	Confirmed DENV	⎼	⎼	Moderate disability	16, M	55	20	65	0.7	NA	+ (5)	+ (3)	⎼	+ (3)	⎼	⎼	⎼	13	⎼
14	Probable JEV	⎼	⎼	Death	37, F	NA	NA	NA	NA	NA	+ (2)	+ (2)	⎼	+ (2)	+ (2)	+ (20)	⎼	9	⎼

HSV-1, herpes simplex virus 1; EBV, Epstein-Barr virus; CMV, cytomegalovirus; EV-D68, enterovirus D68; RV-A, rhinovirus A; DENV, dengue virus; JEV, Japanese encephalitis virus; HIV, human immunodeficiency virus; TB, tuberculosis; NA, not available; +, characteristics present; ⎼, characteristics absent.

^a^Based on Glasgow Outcome Scale: 1 death, 2 persistent vegetative state, 3 severe disability, 4 moderate disability, and 5 low disability

^b^Glasgow Coma Score: 3–8 severe, 9–12 moderate, 13–15 mild.

Thrombocytopenia, the hallmark of dengue fever was not present in the DENV case on admission and throughout the hospitalization based on serial hematological examination as it might not manifest by the time of neurological presentation. The leukocyte count was slightly high in admission but well within the normal range one week after admission, which was not unusual as peripheral leukocytosis was reported in an earlier study [19]. The patient had a history of fever for five days followed by decreased consciousness and general seizure for three days before admission. Other features of dengue fever including retro-orbital pain, rashes, petechiae, and myalgia were not reported. The patient survived with mild cognitive sequelae on discharge. DENV NS1 antigen, an early marker of DENV infection, was negative in the CSF by using SD Bioline Dengue NS1 Ag rapid test kit (Standard Diagnostics, Inc., South Korea). Serum was not tested for DENV NS1 antigen due to insufficient volume. Furthermore, pan-flavivirus RT-PCR was also negative for both CSF and serum samples.

The fatal JEV case identified in this study was negative for DENV IgM and experienced myalgia for 20 days accompanied by high-grade fever, seizure, altered consciousness, and skin rash for two days prior to hospitalization. CSF sample is not available from this JEV patient and an attempt to isolate the virus from the serum was not successful. Furthermore, the two EV cases (EV-D68 and RV-A) identified in this study presented to the hospital without high-grade fever or respiratory symptoms, although the EV-D68 case suffered multiple generalized seizures for 30 days prior to admission. These EV patients were discharged without any cognitive sequelae.

## Discussion

There is an increase in the emergence and spread of neurotropic viruses in the Southeast Asian and Western Pacific regions [20]. However, the etiology of CNS infections remains unknown in 50−70% of the cases worldwide and numerous challenges remain in studying the cause of CNS infections even in resource-rich settings [21]. As in many Southeast Asian countries, there is limited laboratory capacity in Indonesia to diagnose CNS viral infections. In addition, there is lack of standard laboratory diagnostic algorithms and the use of virologic investigations has been inconsistent. Here, we report the detection of CNS viral infection in patients admitted to a referral hospital in Manado, North Sulawesi, Indonesia, using vigorous virology screening methods with a core battery of eight groups of neurotropic viruses. HSV-1 was found to be the most commonly identified agent of CNS infection.

In spite of the small sample size, the finding of HSV-1 as the predominantly identified virus in this study was consistent with others from Vietnam [22], India [23], Norway [24], and Poland [25], while other studies in Vietnam [26] and Thailand [27] reported JEV as the most common identified viral cause of CNS infection. Furthermore, recent studies in China [28] and Italy [29] found EV as the primary identified viral pathogen causing CNS diseases. In this study, the case fatality of those with viral etiology (57.1%) was substantially higher than in the region [30,31], with the majority (62.5%) associated with HSV-1. This highlights the significance of HSV-1 as a cause of fatal CNS infection in Indonesia. In our study, two HSV positive cases, one fatal, were found to co-exist with HIV. Although HSV-1 encephalitis was reported to be uncommon in HIV patients [32], our study highlights the need to diagnose HSV in HIV-associated CNS infection as pharmacotherapy for HSV is available to influence the patient outcome.

A definitive diagnosis of dengue was provided by the detection of anti-DENV IgM in the CSF and serum in one patient. Since IgM does not normally cross the blood-brain barrier, detection of anti-DENV IgM in the CSF is an indication of host immune response to DENV invasion in the CNS [33]. The absence of DENV RNA and NS1 antigen in the CSF is not unexpected as low sensitivity of RT-PCR in CSF has been reported due to a lower viral load [34]. The dengue patient in this study did not exhibit shock, bleeding, or thrombocytopenia, and the patient was discharged with mild cognitive sequelae. The absence of notable dengue clinical manifestations in this patient is not uncommon as one study has reported that 57% of confirmed DENV-infected patients with CNS involvement had no characteristic features of dengue on admission [19]. Although CNS manifestations are rare complications of dengue, it is now recognized as an emerging cause of flavivirus encephalitis [34]. There should be an increased awareness among physicians to include dengue in the differential diagnosis of CNS infection as DENV could be a significantly underreported cause of viral infection of the CNS. Studies have shown that the outcome of dengue encephalitis is variable, with most patients recovering spontaneously in one case series and deaths occurring in others [34].

One case with positive JEV IgM in the serum without available CSF was diagnosed as probable JE encephalitis as arbovirus antibodies tend to persist in the serum for many months after acute infection [35]. Based on nationwide seroprevalence studies, JE is now considered endemic in Indonesia and was already reported to be present in North Sulawesi [4]. JEV numbers could have been low in this study since the study population consisted mainly of adults and JE neurological disease is more common in children [36]. In addition, convalescent samples were not obtained in any of the patients to determine seroconversion.

Enteroviruses are linked with aseptic meningitis and encephalitis worldwide. Two cases of EV (EV-D68 and RV-A) were identified by PCR from throat swabs which are known to have higher yields than CSF for EV CNS infection as viral concentration could be low early after illness onset [37]. Our pan-enterovirus PCR primers were designed to detect a conserved segment in untranslated region of EVs and have been successfully used to detect a broad range of EVs including coxsackievirus B3 and rhinovirus C [15,38]; enterovirus A71, rhinovirus A, echovirus, poliovirus, coxsackievirus B4, A6, and A16 [39]; as well as a number of novel enteroviruses, from various types of clinical samples [16,40]. The detection rate could be low in our study population of adults as enterovirus CNS infection is commonly seen in pediatric patients but in less than 1% of adults. Although EV-D68 has been more associated with mild to severe upper and lower respiratory illnesses, some studies have associated this virus with CNS diseases [41,42]. Furthermore, while RV-A is considered the most common cause of upper respiratory tract infection, occasional detection of RV-A from CNS infection has been reported [37,43]. Our RV-A case could be a coexisting infection without sufficient evidence to establish a causal relationship with the CNS infection.

Despite the interesting findings, a high proportion of cases (81.0%) remained undiagnosed for viral etiology as with other regional studies [22,26,27,30,31]. This could be due to the timing of specimen collection, limited viral IgM serology panels, unavailability of convalescent serum, non-viral non-infectious causes, immune-mediated causes, or infection due to novel pathogens. Earlier studies have shown the usefulness of serology since viremia might have waned to undetectable levels by the time of clinical presentation [44].

There are several limitations in our study. Firstly, serology is limited to that of arboviruses, and the use of HSV IgM might allow more cases to be diagnosed [45]. Second, not all potential causes of viral meningitis/encephalitis were screened in this study; influenza virus, rubella virus, adenovirus, parechovirus, and lymphocytic choriomeningitis virus may have been missed due to lack of testing. Third, the incidence of EV might have been underestimated in this study due to failure to collect swab specimens in 48 patients (64.9%). Forth, EBV and CMV may remain latent in B cells and can make PCR positive results difficult to interpret [46]. Fifth, MRI which is more sensitive than CT, was not performed under resource-limited healthcare settings. Sixth, the prevalence of etiological agents detected in this study might be low compared with other studies [25,26,28,29,47], possibly due to the different molecular tools used (pan-family vs virus-specific PCR/RT-PCR) and case selection methods (suspected CNS infection vs encephalitis/meningoencephalitis only). Seventh, with a small sample size, our conclusions on viral etiology might not be statistically significant. Finally, CSF specimens were not always available to make a definitive diagnosis of viral etiology as some patients did not consent to lumbar puncture and viral transport medium for throat swabs was not always available. However, even with these limitations, a range of viruses was identified as associated with CNS disease in eastern Indonesia suggesting that testing should be broader than is routinely conducted.

## Conclusions

Very little has been done in Indonesia to systematically survey patients admitted to hospitals with CNS infections. Many challenges exist in determining the etiology of viral CNS infections including the timing of specimen collection, optimal storage, locating the laboratory network, and cost of testing. Determining the causes of CNS infections is becoming increasingly important with the emergence of new viruses. With the challenge of taking multiple samples in general hospitals, CSF should be emphasized as the recommended specimen on admission for patients presenting with suspected CNS infection with a follow-up serum specimen at discharge (or death) for serologic confirmation. The results of this study highlight the importance of using a wide range of molecular panels and detection methods for CNS viruses as to optimize treatment strategies, guide public health measures or develop targeted vaccination recommendations. However, in resource-limited settings, it is highly recommended that initial viral screening should include HSV and EV as well as flavivirus serological assays.

## Supporting information

S1 Fig Representative gel electrophoresis with positive result of pan-enterovirus RT-PCR and pan-herpesvirus PCR. Amplified PCR products were visualized on 1.5% agarose gel electrophoresis stained with SYBR Safe DNA Gel Stain (Thermo Fisher Scientific, Waltham, MA, USA).
(PDF)
