# Supplementary material for: Detection of central nervous system viral infections in adults in Manado, North Sulawesi, Indonesia
Source: PLoS One. 2018 Nov 16;13(11):e0207440. doi: 10.1371/journal.pone.0207440 (PMC6239303; doi:10.1371/journal.pone.0207440)
Supplement: S1 Fig — Amplified PCR products were visualized on 1.5% agarose gel electrophoresis stained with SYBR Safe DNA Gel Stain (Thermo Fisher Scientific, Waltham, MA, USA). (PDF) [file pone.0207440.s001.pdf]

Supporting Information 1 Figure

Pan-enterovirus RT-PCR

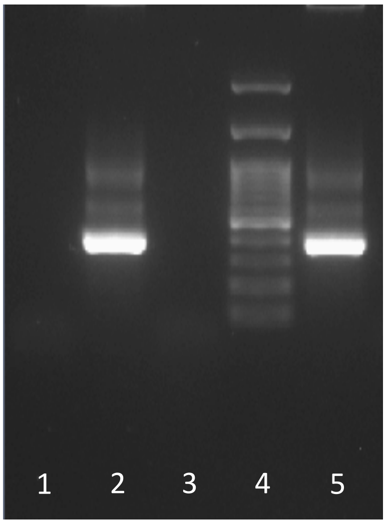

1. Unknown sample #1
2. Unknown sample #2 with enterovirus-specific band. Band was purified and sequenced, from which enterovirus D68 was identified.
3. Negative control
4. DNA ladder
5. Positive control

Pan-herpesvirus PCR

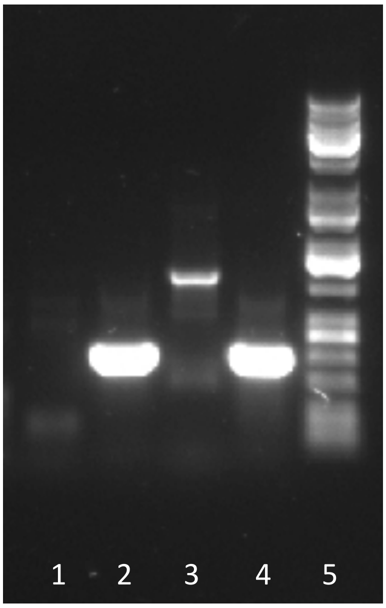

1. Negative control
2. Unknown sample #1 with herpesvirus-specific band. Band was purified and sequenced, from which HSV-1 was identified.
3. Unknown sample #2 with non-herpesvirus specific band
4. Positive control
5. DNA ladder
